# High Efficiency Dual-Band Dual-Circularly Polarized Transmitarray Antenna

**DOI:** 10.3390/mi16030260

**Published:** 2025-02-26

**Authors:** Tianling Zhang, Boxiang Yang, Jiayin Guo, Yuanjun Shen, Liangqin Luo, Lei Chen

**Affiliations:** 1 School of Electronic Engineering, Xidian University, Xi’an 710071, China; yuanjun.shen@xidian.edu.cn (Y.S.); lqluo@stu.xidian.edu.cn (L.L.);; 2 Guangzhou Institute of Technology, Xidian University, Xi’an 710071, China; ted5o1@163.com; 3 State Key Laboratory of Mobile Network and Mobile Multimedia Technology, Department of RCH System, ZTE Corporation, Xi’an 710061, China

**Keywords:** dual band, dual circularly polarized, transmitarray antenna, high efficiency

## Abstract

A dual-band dual-circularly polarized transmitarray antenna (TA) operating in the 28/39 GHz millimeter-wave band is proposed in this article. The TA unit consists of two parts: a broadband linearly polarized (LP) receiving part and a dual-band dual-circularly polarized transmitting part. An over-2-bit phase compensation is achieved by changing the size of the U-shaped slot and the rotation status of the receiving part. A 24 × 24 TA model with an aperture size of 88.8 mm × 88.8 mm is built up by using the proposed units and fed by a wide-band corrugated horn antenna. The simulated results show that the maximum gain of the dual-band dual-circularly polarized TA is 26.28 dBic within the low-band (26.5–29.5 GHz) and 27.4 dBic within the high-band (37–40 GHz). To verify the accuracy of the simulation, a prototype of the proposed TA is fabricated and measured. The measured maximum efficiencies are 53.56% and 42.89% in low and high bands, respectively. The proposed TA covers two bands (28/39 GHz) for fifth generation (5G) millimeter-wave applications. Moreover, it features low cost, high gain, and high efficiency.

## 1. Introduction

In recent years, millimeter-wave wireless communication technology has garnered widespread attention due to its rich spectrum resources. Millimeter-wave technology extends microwave wireless communication technology into higher frequency bands and has become essential for the development of many emerging technologies [1,2]. In current applications, such as navigation satellites, radio-frequency identification (RFID), and 5G communication, circularly polarized antennas are indispensable. Compared to LP antennas, circularly polarized antennas have advantages such as eliminating the need for alignment between transmitting and receiving antennas, and providing improved resistance to multi-path interference. Thus, they are more suitable for communication systems requiring high precision and interference resistance. At present, much research has been conducted on circularly polarized antennas [3,4,5], revealing advantages such as low radar cross-section (RCS) and ultra-broadband, enabling the multi-functionalities of circularly polarized antennas and greatly enriching their application scenarios. However, with the growing need for high-frequency, multi-band communication and complex electromagnetic environmental applications, more requirements are being placed on the functionality and characteristics of antennas, and traditional single-characteristic antennas will no longer be adequate. Specifically, high-gain multi-band antennas remain an urgent issue that needs to be addressed.

In recent years, scholars have made progress in their research into transmitarray antennas for their low cost, simple structure, flexible design, and high gain. TAs have been widely used in satellite communications, 5G and future 6G point-to-point communications, and radar systems, making them a key focus in antenna technology research. In practical applications, LP transmitarray antennas are used based on their fundamental design, due to their simplicity and ease of optimization, and LP TAs are widely used across various fields. For instance, in wireless communication systems, LP TAs enable high gain beam steering through precise phase control [6,7,8,9]. In satellite communication systems, LP TAs can also emit multiple beams through phase control, enabling spatial isolation to achieve frequency reuse and polarization multiplexing. This approach significantly enhances the capacity of communication satellites [10,11,12]. As communication systems become complex, circularly polarized (CP) transmitarray antennas have garnered great interest because of their ability to provide stable communication links, high gain, and enhanced performance in mitigating multipath distortion and polarization mismatch [13,14,15,16]. However, as modern communication systems demand multi-band operation, high spectral efficiency, and multifunctionality, conventional single-frequency and single-polarization TAs no longer to satisfy these requirements. Dual-band, dual-circularly polarized transmitarray antennas have emerged as a key research focus due to their unique capabilities.

The technology for achieving dual-band dual-circularly polarization is generally realized through two approaches. The first is the folded antenna design. A folded antenna uses two array surfaces, with electromagnetic waves reflecting multiple times between the two surfaces before radiating into free space [17,18,19,20]. This method effectively increases freedom in the design, making it easier to implement complex functions, such as dual circularly polarized or multi-beam patterns. The disadvantages of this type of antenna are also evident, such as multiple reflections leading to higher losses, which in turn reduce antenna efficiency. Additionally, the use of multilayer structures results in higher manufacturing costs. The second approach is the traditional transmitarray design [21,22,23]. In [21], the TA used a receive-transmit form employing two kinds of LP units, which operate in two frequency bands, respectively. The staggered arrangement of two units forms a TA, receiving an LP wave and converting it into a dual-band dual-circularly polarized wave by rotating the transmitting patch. However, this decomposition results in a 3 dB gain loss in overall antenna efficiency. In [23], a TA unit is constructed using the form of an antenna–filter–antenna (AFA) structure and implements dual-band operation using a single unit structure. Following the approach in [21], dual-circularly polarized characteristics at both working bands are achieved by decomposing the LP wave into a circular polarization wave. Similarly, the antenna efficiency of the proposed TA is only about 20%.

Furthermore, TA unit dimensions are closely related to its operating wavelength, and the unit design needs a half-wavelength periodicity. Moreover, signal transmission in the millimeter-wave band is susceptible to atmospheric absorption and rain fade, which imposes higher requirements on antenna materials and manufacturing processes [24,25,26,27,28,29]. The printed circuit board (PCB) manufacturing process is a good candidate for fabricating millimeter-wave antennas due to its high accuracy and multi-layer lamination. By utilizing PCB processes, the dimension of the copper pattern can be etched with a tolerance of ±0.02 mm. Moreover, various surface treatment processes are available with PCB. In addition, when the drilled hole diameters are greater than 0.2 mm, the maximum aspect ratio is 22:1 and mechanical drilling can achieve a minimum diameter of 0.1 mm. As reported in [27], a compact Ka-band antenna array was proposed, featuring a multilayer structure composed of a driven slot patch layer and a parasitic patch array layer. The antenna was fabricated using a PCB lamination process. Due to the high precision of PCB manufacturing, the antenna array achieved miniaturization, wide bandwidth, and high gain, making it well-suited for millimeter-wave wireless communication systems. Additionally, in [28], an air-filled substrate-integrated waveguide (AF–SIW) CP antenna array is presented. The antenna array comprises three layers of PCB, which form the AFS–IW feeding network, and one layer of aluminum, which serves as the metal cavity radiation element. The entire antenna was fabricated using PCB lamination techniques, and the measured results demonstrate a wide axial ratio (AR) bandwidth and excellent input impedance matching.

To solve the current issue of low antenna efficiency in TA design, a dual-band dual-circularly polarized TA in a receiver–transmit form is proposed and the PCB fabrication process was employed in its manufacture. The thinnest part of the copper pattern of the TA unit is 0.13 mm. The receiving part of the proposed TA unit is a broadband LP patch antenna, and the transmitting part is a dual-band dual-circularly polarized patch antenna. By changing the length of the U-slot of the receiving patch, a continuous 90° phase shift can be achieved. When the receiving patch is rotated by 180°, a 1-bit phase change occurs. By combining these two methods, over-2-bit phase quantization compensation can be realized. Current dual-band dual-circularly polarized TAs mainly require two different type units, which not only decreases the antenna efficiency, but also increases the complexity of the design. This work breaks through these limitations by using a single unit to achieve dual-band dual-circularly polarized radiation characteristics. The measured results show that the maximum efficiency of the antenna is above 40% in both the low and high bands, indicating that the proposed dual-band dual-circularly polarized TA can more effectively utilize the radiation aperture and improve antenna efficiency. Due to the high antenna efficiency, the proposed TA can be used in 5G/6G millimeter-wave communication systems or millimeter-wave radar systems.

The subsequent structure of the articles is as follows. Section 2 explains the proposed TA unit’s detailed working principle and design procedure. In Section 3, the simulation and measurement results are compared and discussed. Finally, Section 4 presents the conclusion.

## 2. Theory and Design

### 2.1. Analysis of the Radiation Characteristics of the TA Unit

**Figure 1:**
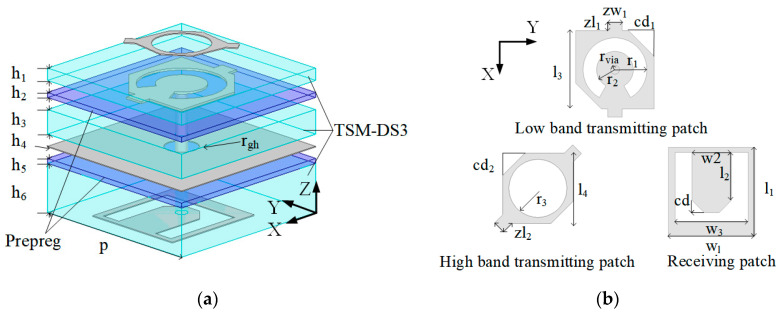
Geometry of dual-band dual-circularly polarized unit: (**a**) 3D view; (**b**) Radiation patches of the unit (h_1_ = 0.254 mm, h_2_ = 0.104 mm, h_3_ = 0.508 mm, h_4_ = 0.018 mm, h_5_ = 0.104 mm, h_6_ = 1.016 mm, zw_1_ = 0.4 mm, zl_1_ = 0.23 mm, cd = 0.34 mm, cd_1_ = 0.715 mm, cd_2_ = 0.44 mm, l_3_ = 2.18 mm, l_4_ = 1.3 mm, zw_2_ = 0.24 mm, zw_2_ = 0.24 mm, w_1_ = 2.64 mm, w_2_ = 1.27 mm, w_3_ = 2.3 mm, l_1_ = 2.56 mm, l_2_ = 1.1 mm, r_gh_ = 0.35 mm r_via_ = 0.125 mm, r_1_ = 0.89 mm, r_2_ = 0.49 mm, r_3_ = 0.57 mm, *p* = 3.7 mm).

The dual-band dual-circularly polarized transmitarray unit is designed with a stacked structure. The structure of the unit is shown in Figure 1, composed of four copper layers, two layers of prepreg (FR27-0040-43F, εr = 2.79, and tanδ = 0.0014), and three layers of substrates (TSM-DS3, εr = 3, and tanδ = 0.0011). The operational principle of the proposed unit involves the LP waves emitted by the feed antenna. Then, it transfers the low-band energy to the low-band transmitting patch through the metallic route, subsequently radiating to free space via RHCP waves. Similarly, the high-band energy propagates to the high-band transmitting patch, subsequently radiating to free space with LHCP waves. Due to the U-shaped slot, the resonance point of the antenna unit is increased, so that the working frequency band of the linearly polarized unit can cover the two design working bands.

**Figure 2:**
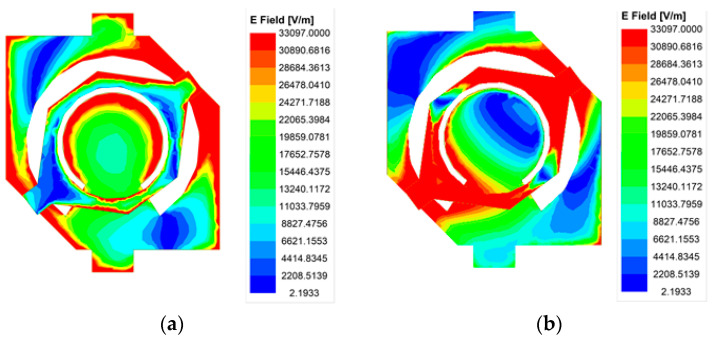
The surface electric field distribution on the radiation patch of the proposed TA unit at (**a**) 28 GHz; (**b**) 38 GHz.

Next, the working mechanism of the transmitting patch is analyzed. First, the low-band transmitting patch is located beneath the high-band transmitting patch. This radiating patch is a square patch with removal of its center circular part and the addition of two branches, and its opposite corners are chamfered. This patch emits RHCP waves in the low band. The high-band transmitting radiation patch is located on the top layer of the unit, which is a circular ring with short branches on both sides. When the high-band transmitting patch receives the electromagnetic energy propagated by the receiving patch, it is sufficiently excited to generate LHCP electromagnetic waves. The electric field distribution of the radiating patch is shown in Figure 2. In the low band, the electric field is almost nonexistent on the high-band transmitting patch, with the field primarily concentrated around the lower-band transmitting patch. In contrast, in the high band, the electric field is stronger around the high-band transmitting patch, while the field distribution around the lower-band transmitting patch is much weaker. The stacked structure realizes the dual-band dual-circularly polarized functionality within an antenna unit. The unit efficiently utilizes the radiation aperture, enhancing antenna efficiency across both frequency bands.

### 2.2. Basic Theory and Performance of the Unit

Due to the unique feeding mechanism of the TA, the unit must compensate for the phase delay introduced by the feed antenna at the surface. As outlined in the literature [30], the phase compensation technique results in a 3 dB loss for 1-bit phase quantization and a 0.6 dB loss for 2-bit phase quantization. Therefore, to ensure high efficiency for the TA, it is better to use more than two-bit quantization or continuous phase compensation.

When the beam is directed as normal to the surface, the phase compensation required for each unit in the array is calculated by Equation (1):

(1) $$\Phi (x_{i},y_{i})=k_{0}d_{i}$$

where $d_{i}$ is the distance from the phase center of the feed to the center of each unit, and $k_{0}$ is the number of waves in free space. The over-2-bit phase compensation can be quantified by Equation (2):

(2) $$\left\{\begin{array}{l} 0{}^{\circ}\leq \varphi <90{}^{\circ}\to \varphi =[0{}^{\circ},90{}^{\circ}]\\ 90{}^{\circ}\leq \varphi <180{}^{\circ}\to \varphi =90{}^{\circ}\\ 180{}^{\circ}\leq \varphi <270{}^{\circ}\to \varphi =[180{}^{\circ},270{}^{\circ}]\\ 270{}^{\circ}\leq \varphi <360{}^{\circ}\to \varphi =270{}^{\circ} \end{array}\right.$$

The phase compensation method in this paper can be illustrated in two steps. In the first step, the receiving patch of the TA unit is rotated 180°, which means that the statement of the receiving patch is changed from State A to State B. Thus, two quantization phases of 1-bit of 0° and 180° are generated with State A and B. Then, the second step is to adjust the length of the U-slot of the receiving patch of the dual-band dual-circularly polarized TA unit to produce a continuous phase shift of 0–90°, so as to realize the over-2-bit phase shift ranges of 0–90° and 180–270°.

**Figure 3:**
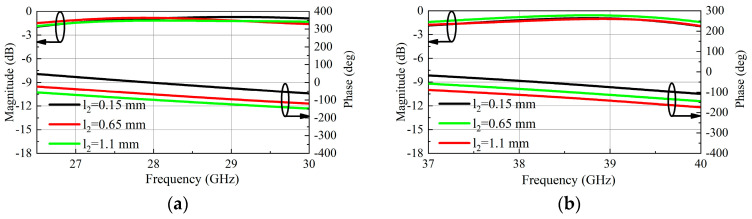
Simulated transmission magnitudes and phases as a function of l_2:_ (**a**) Low-band; (**b**) High-band.

**Figure 4:**
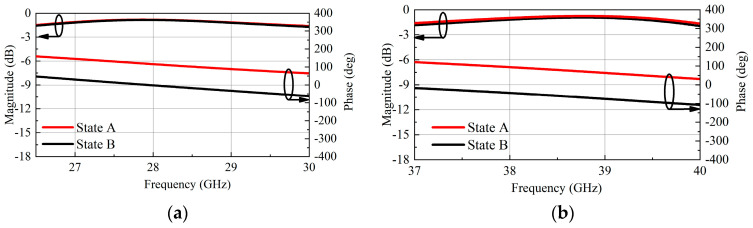
Simulated transmission magnitudes and phases of two states of the unit: (**a**) Low-band; (**b**) High-band.

To verify the theoretical analyses and further explore performance, simulation of the TA unit is conducted by using HFSS. When the length parameter l_2_ of the U-slot is changed, the transmission phase also changes. The curves of transmission amplitude and phase varying with l_2_ are shown in Figure 3. When l_2_ is changed from 0.15 mm to 1.1 mm, the transmission coefficient is greater than −1.6 dB in the low band, the phase shifting range is 90°. In the high band, the transmission magnitude is higher than −1.8 dB, and the phase shift range is 90°. Figure 4 illustrates the transmission amplitude and phase of the unit under State A and State B, where a phase shift of 180° is generated and the transmission amplitude remains essentially consistent. By combining these two-phase shift methods, a phase shift range of over-2-bit can be obtained, so as to achieve a smaller phase quantization error than that of 2-bit.

**Figure 5:**
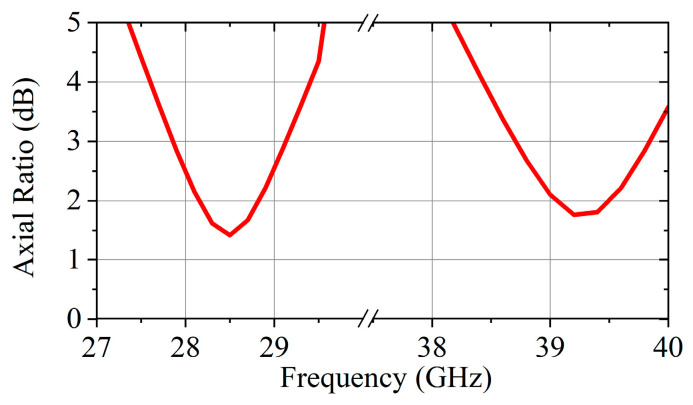
The simulated axial ratio of the proposed TA unit.

**Figure 6:**
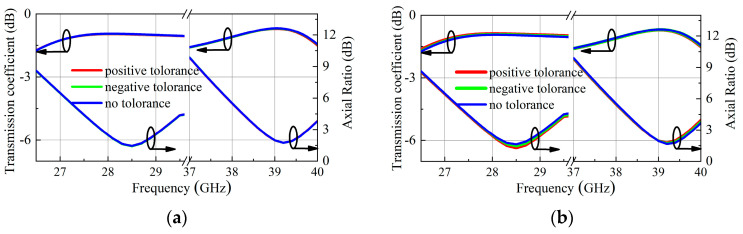
Simulated transmission coefficient and axial ratio of the proposed TA unit: (**a**) variation zl_1;_ (**b**) variation w_1_.

The simulation axial ratio result of the unit is shown in Figure 5. At 28.75 GHz and 39 GHz, the axial ratio is less than 2 dB, and the 3-dB AR bandwidth is 1.1 GHz and 1 GHz, respectively. This result indicates that the proposed unit can efficiently radiate LHCP electromagnetic waves in the high band and RHCP electromagnetic waves in the low band. In order to verify the manufacturing feasibility, a PCB fabrication process tolerance analysis was performed for both zl_1_ and w_1_ parameters, and the simulated transmission coefficient and the axial ratio of the proposed unit are shown in Figure 6. According to Figure 6, the radiation performance of the unit is very stable under a tolerance of ±0.02 mm.

## 3. Measurement and Simulation Discussion

**Figure 7:**
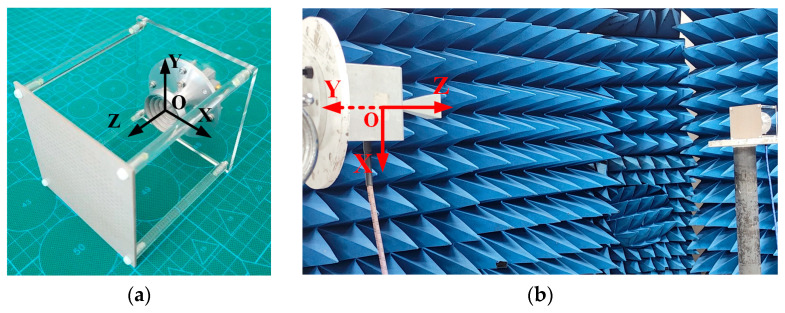
Dual-band dual-circularly polarized TA: (**a**) Prototype; (**b**) Under test.

The simulation of the proposed TA is accomplished by using finite-element boundary-integral equations (FEBIs) in Ansys HFSS, and the fabricated prototype is shown in Figure 7a. The E-plane and H-plane of the feed antenna are the XOZ plane and the YOZ plane of the Cartesian coordinate system, respectively. In Figure 7b, the measurement setup is shown. The proposed TA includes 24 × 24 units and diameter of the circular aperture is 88.8 mm.

Typically, TAs use horn antennas as feed antennas. Thus, corrugated horn is employed in this case, which uses corrugated structures at the aperture to suppress sidewall currents, effectively reducing the impact of diffraction fields on radiation characteristics [31]. By optimizing the corrugation spacing and slot depth, beam symmetry between the E-plane and H-plane within the 10 dB beamwidth was improved. Therefore, the designed corrugated horn radiates most energy on the transmitarray surface. The radiating beam of the proposed TA is designed to be normal to the surface.

**Figure 8:**
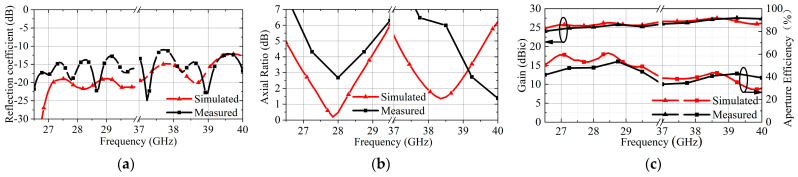
Simulated and measured results: (**a**) Reflection coefficient, (**b**) Axial Ratio, (**c**) Antenna efficiency and gain of the proposed TA.

Next, Figure 8 presents the simulated and measured results of the proposed TA, including reflection efficient, gain, antenna efficiency, and axial ratio. From Figure 8a, it can be observed that the reflection coefficient of the antenna is below −10 dB within the low and high working bands for both simulated and measured results, indicating good impedance matching characteristics. From Figure 8b, the measured results reveal that the axial ratio performance deteriorates in the low working band, while in the high working band the axial ratio shifts toward higher frequencies. The deterioration of the axial ratio makes increases TA in the cross-polarization level and decreases it in the measured gain. These phenomena are due to fabrication errors. This observation is consistent with the trends in gain and antenna efficiency shown in Figure 8c. The simulated gain reaches a maximum of 26.3 dBic at 28.45 GHz in the low band and 27.48 dBic at 38.5 GHz in the high band. The measured maximum gain is 25.83 dBic at 28.75 GHz in the low band and 27.57 dBic at 39.25 GHz in the high band. The antenna efficiencies can be calculated by

(3) $$\eta_{eff}=\frac{G\lambda^{2}}{4\pi A}$$

where *A* is the area of the transmitarray aperture, and in this work the *A* is 6193 mm^2^. *G* is gain and *λ* is the wavelength corresponding to *G*. Therefore, the simulated maximum antenna efficiency in low band and high band is 60.1% and 43.7%, and the measured maximum antenna efficiency in low band and high band is 53.56% and 42.89%.

**Figure 9:**
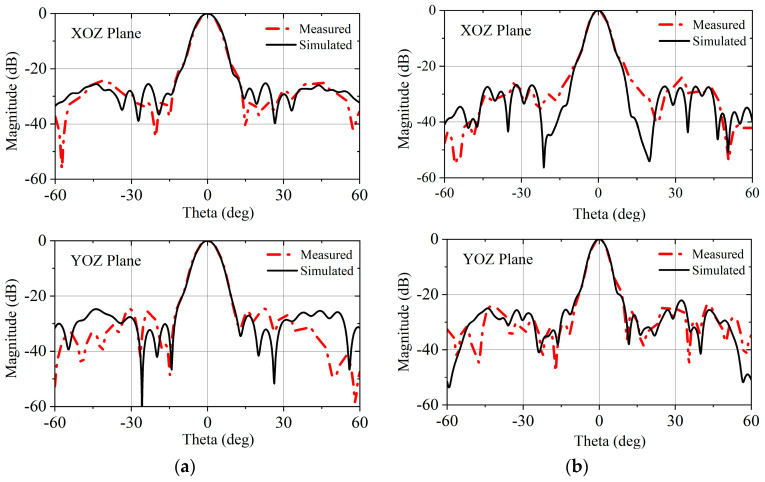
Simulated and measured normalized co-polarization radiation patterns of TA. (**a**) 28 GHz; (**b**) 38.5 GHz.

Figure 9a,b presents the normalized co-polarization radiation patterns of the dual-band dual-circularly polarized TA in XOY and YOZ planes at 28 GHz and 38.5 GHz, respectively. The sidelobe level (SLL) is below −20 dB and the symmetry between the XOY and YOZ plane patterns is good. By comparing the normalized co-polarization radiation patterns obtained from simulation and measurement, it can be observed that the shapes of the main lobes in both patterns match very well, which also validates the accuracy of the simulation.

Table 1 summarizes the performance of the reported and the proposed TAs. It shows that the antenna efficiency of the proposed TA is higher than that of the reported. Compared with TA in [21], the proposed dual-band dual-circularly polarized TA emits RHCP and LHCP waves directly, foregoing the need to split LP wave into two circularly polarized waves. As a result, antenna efficiency is higher. Structurally, the proposed design employs a simplified four-metal layer structure, which not only reduces fabrication complexity compared to the six–seven layer designs reported in Table 1, but also minimizes potential losses while maintaining superior performance. In [32], a linearly polarized microstrip patch antenna is used as the receiving antenna, and a shaped single-arm spiral antenna is selected as the transmitting antenna. The TA in [32] achieves antenna efficiency of 60% in a single band due to 3-bit phase compensation. Although the antenna efficiency of the proposed TA in this work is slightly lower than that of the TA in [32], it can work in dual band with different circular polarization.

Overall, this work combines high efficiency and a simple structural design. Moreover, during the simulation process, the results showed that the oblique incidence performance of the unit remains stable. Furthermore, it can be predicted that, by moving the position of the feed antenna, the TA can achieve beam scanning capability, which is required in future millimeter-wave radar systems.

## 4. Conclusions

In this article, an over-2-bit dual-band dual-circularly polarized TA is proposed. Through the dual-band dual-circularly polarized transmitting patch and the wideband linearly polarized receiving patch, the proposed TA can work in two frequency bands at the same time, receive the linear polarized wave, and transmit the LHCP wave and the RHCP wave. A TA with the thinnest part of the transmitting and receiving patches, i.e., 0.13 mm, is manufactured by using the PCB fabrication process. The measured results show that the maximum antenna efficiency of both working bands is above 40%, which proved that the precision PCB process is very suitable for making millimeter-wave band TAs. Currently, some microstrip antennas working in the terahertz band are manufactured using the PCB fabrication process due to its high processing accuracy. Due to the simple structure of the proposed TA, it is believed that it can be extended to work in higher frequency bands by using the PCB fabrication process in the future.

## Figures and Tables

**Table 1:** Comparison with the Proposed and Reported Transmitarray Antennas.

Ref.	Metal Layers	Antenna Features	Frequency (GHz)	Antenna Efficiency (%) ^1^	Gain (dBic)	Phase Range
[17]	6	Single band Dual-CP	27	26.6/20.9	21.4/20.3	cons
[19]	6	Dual band Dual-CP	11.6/14.2	23/11.7	24.27/23.08	2-Bit
[21]	3	Dual band Dual-CP	12/14.2	32.2/28.9	23.9/24.5	cons
[22]	5	Single band Dual-CP	20	25.8/26.4	23.6/23.1	2-Bit
[23] ^2^	7	Dual band Dual-CP	18/28 19/29	26.2/15.6 24.4/15.2	27/27 25/25	cons
[32]	3	Single band Single-CP	20	60.1	32.1	3-Bit
This Work	4	Dual band Dual-CP	28/39	53/42	25.2/26.1	Over-2-Bit

^1^ The formula for calculating antenna efficiency is $G\lambda^{2}/4\pi A$. ^2^ The values in the first/second row indicate results in the low-band/high-band, while the values before/after “/” represent results for the RHCP/LHCP beam.

## Data Availability

The datasets presented in this article are not readily available because the data are part of an ongoing study. Requests to access the datasets should be directed to ted5o1@163.com.

## References

[1] Rappaport T.S., Sun S., Mayzus R., Zhao H., Azar Y., Wang K., Wong G.N., Schulz J.K., Samimi M., Gutierrez F. (2013). Millimeter Wave Mobile Communications for 5G Cellular: It Will Work!. IEEE Access.

[2] Yang B., Yu Z., Lan J., Zhang R., Zhou J., Hong W. (2018). Digital Beamforming-Based Massive MIMO Transceiver for 5G Millimeter-Wave Communications. IEEE Trans. Microw. Theory Tech..

[3] Chen W.H., Ye L.H., Ding K., Jiang F., Wu D.-L. (2024). A Broadband Circularly Polarized Antenna Using Characteristic Mode Analysis. IEEE Trans. Antennas Propag..

[4] Wang K.X., Teng W., Chen Z., Wong H., Zhang Q. (2024). Design of an Ultrabroadband Circularly Polarized 3-D-Printed Millimeter-Wave Lens Antenna. IEEE Trans. Antennas Propag..

[5] Guo K., Sun X., Su T., Wu B. Broadband Circularly Polarized Antenna Based on Cross-Fed Configuration. Proceedings of the 2023 IEEE 11th Asia-Pacific Conference on Antennas and Propagation (APCAP).

[6] Xue L., Xu S., Wang M., Yang F., Li M. (2024). Wideband Reconfigurable Transmitarray Antenna Using Tightly Coupling Effect With Wide-Angle Beam Scanning Capability. IEEE Antennas Wirel. Propag. Lett..

[7] Zhu L., Guo X., Sun S., Wu W. (2024). Beam-Scanning Transmitarray With Extended Wide Scanning Range. IEEE Antennas Wirel. Propag. Lett..

[8] Meng X., Wang Y., Zhang H., Wu T., Zhang A., Chen X. (2024). A Double-Layer Metal-Only Transmitarray for Beam Scanning. IEEE Antennas Wirel. Propag. Lett..

[9] Chen T., Song L., Liu Y. (2024). A Broadband Dual-Polarized Reconfigurable Transmitarray With Sum and Difference Beam-Steering Capabilities. IEEE Trans. Antennas Propag..

[10] Song L., Qin P., Du J., Guo Y.J. Multi-Beam Conformal Transmitarray Synthesis for Advanced Wireless Systems. Proceedings of the 2023 17th European Conference on Antennas and Propagation (EuCAP).

[11] Xi B., Xue Q., Bi L., Wang Y. Design of Multi-Beam Transmitarray Antenna Using Alternating Projection Method. Proceedings of the 2018 IEEE International Conference on Computational Electromagnetics (ICCEM).

[12] Zeng L., Qin F., Zhang H. Bidirectional Multi-Beam Transmit-Folded-Transmit Antenna Based on Polarization Rotating Metasurface. Proceedings of the 2022 IEEE 10th Asia-Pacific Conference on Antennas and Propagation (APCAP).

[13] Wei F., Hao J.-W., Xu L., Shi X. (2021). A Circularly Polarized 3-D Printed Dielectric Transmitarray Antenna at Millimeter-Wave Band. IEEE Antennas Wirel. Propag. Lett..

[14] Dai X.W., Li Z., Ruan H., Yu W., Liu L., Luo G.Q. (2024). An Ultralow-Profile Folded Transmitarray Antenna with Both-Sides Beam Regulate for K-Band Circularly Polarized OAM Waves. IEEE Antennas Wirel. Propag. Lett..

[15] Li T.-J., Wang G.-M., Li H.-P., Hou H.-S. (2022). Circularly Polarized Double-Folded Transmitarray Antenna Based on Receiver-Transmitter Metasurface. IEEE Trans. Antennas Propag..

[16] Yang W., Tang K., Zhu Y., Chen K., Zhao J., Jiang T., Feng Y. (2024). Wideband Dual-Circularly Polarized Transmit-Reflect-Array Antenna with Energy Allocation Based on Hybrid Metasurface. IEEE Trans. Antennas Propag..

[17] Liu W., Li S., Chen L., Zhang C., Deng L. (2024). A Broadband High-Efficiency Spin-Decoupled Folded Transmitarray Antenna With Independent Beam Control. IEEE Trans. Antennas Propag..

[18] Zhang P.-P., Zhu X.-C., Hu Y., Hao Z.-C., Luo G.-Q. (2022). A Wideband Circularly Polarized Folded Reflectarray Antenna With Linearly Polarized Feed. IEEE Antennas Wirel. Propag. Lett..

[19] Lei H., Liu Y., Jia Y. Dual-Band Dual-Circularly Polarized Folded Transmitarray Antenna Based on Metasurface. Proceedings of the 2021 International Conference on Microwave and Millimeter Wave Technology (ICMMT).

[20] Lei H., Liu Y., Jia Y., Zhong Y., Liu Z.-X. (2024). A Low-Profile 2-D Beam-Scanning Circularly Polarized Antenna Combining Reflectarray and Transmitarray. IEEE Antennas Wirel. Propag. Lett..

[21] Cai Y.-M., Li K., Li W., Gao S., Yin Y., Zhao L., Hu W. (2020). Dual-Band Circularly Polarized Transmitarray With Single Linearly Polarized Feed. IEEE Trans. Antennas Propag..

[22] Hu J., Wang C., Deng M., Deng L., Wong H., Sang L. (2024). A Dual-Channel Linear-to-Circular Polarization Conversion Transmitarray With Independent Wavefront Control Capability. IEEE Trans. Microw. Theory Tech..

[23] Tong X., Zeng W., Li Y., Wu F., Jiang Z.H., Sauleau R., Hong W. (2024). A Dual-Band Dual-Circularly Polarized Transmit-Array Antenna and Its Application for Four-Color Scheme Multibeam Generation. IEEE Trans. Antennas Propag..

[24] Perdigones F., Quero J.M. (2022). Printed Circuit Boards: The Layers’ Functions for Electronic and Biomedical Engineering. Micromachines.

[25] Shi J., Xu R., Wu B., Wang L., Jiang R. (2024). A Wideband Millimeter-Wave Dual-Beam Dielectric Resonator Antenna with Substrate Integration Capability. Micromachines.

[26] Ghouse P.S.B., John D.M., Mane P.R., Saha D., Balavalikar Shivarama S., Pathan S., Raghavendra Bhat B., Vincent S., Ali T. (2024). A Compact MIMO Antenna Based on Modal Analysis for 5G Wireless Applications. Micromachines.

[27] Deng K., Zhang N., Yang G., Li Y., Song R., Liu N. (2024). A Compact Millimeter-Wave Multilayer Patch Antenna Array Based on a Mixed CPW-Slot-Couple Feeding Network. Micromachines.

[28] He W., Hong J., Ren Y., Deng Y., Wang X., Fang X. (2024). A High Gain Circularly Polarized Slot Antenna Array for 5G Millimeter-Wave Applications. Sensors.

[29] Cao Y., Zhang M., Fan C., Chen J.-X. (2024). A Broadband Transmitarray Antenna Using a Metasurface-Based Element for Millimeter-Wave Applications. Micromachines.

[30] Yang H., Yang F., Xu S., Li M., Cao X., Gao J., Zheng Y. (2017). A Study of Phase Quantization Effects for Reconfigurable Reflectarray Antennas. IEEE Antennas Wirel. Propag. Lett..

[31] Olver A.D., Clarricoats P.J., Kishk A.A., Shafai L. (1994). Microwave Horns and Feeds.

[32] Zheng B., Fan Y., Cheng Y.J. (2023). Wideband High-Efficiency Circularly Polarized Transmitarray with Linearly Polarized Feed. IEEE Antennas Wirel. Propag. Lett..

